# Beyond immune activation: Nitric oxide‐mediated tumour microenvironment remodeling operates as a new paradigm for cancer immunotherapy

**DOI:** 10.1002/ctm2.70794

**Published:** 2026-08-31

**Authors:** Xie Xinxin, Xu Shuyu, Liang Xiaolong

**Affiliations:** ^1^ Beijing Key Laboratory of Ultrasound Targeted Drug Delivery and Frontier Imaging Technology State Key Laboratory of Vascular Homeostasis and Remodeling Department of Ultrasound Peking University Third Hospital Beijing People's Republic of China; ^2^ Beijing Key Laboratory of Interdisciplinary Research in Gastrointestinal Oncology (BLGO) Peking University Third Hospital Beijing People's Republic of China

1

Cancer immunotherapy has fundamentally revolutionized oncology by harnessing the host immune system to eradicate malignant cells. Immune checkpoint blockade (ICB), particularly antibodies targeting the PD‐1/PD‐L1 axis, has achieved remarkable clinical success across a spectrum of cancer types.^1^ However, durable responses remain restricted to a minority of patients, notably those with immunologically ‘cold’ tumours. Accumulating evidence indicates that therapeutic resistance stems not merely from a deficit in immune activation, but rather from a hostile tumour ecosystem governed by aberrant vasculature, severe hypoxia, physical immune exclusion and profound immunosuppression. Consequently, the next generation of cancer immunotherapy demands a conceptual evolution: transitioning from the conventional focus on single‐cell immune activation toward the active remodeling of the tumour microenvironment (TME) to engineer an immune‐permissive ecosystem.

Nitric oxide (NO), a small and highly reactive signalling molecule, has long been appreciated for its diverse biological repertoire, ranging from vascular tone regulation to immune modulation and cytotoxicity. Historically, NO was predominantly investigated as a direct antitumour agent capable of inducing lethal oxidative and nitrosative stress. Yet, emerging insights reveal that the functional scope of NO extends far beyond direct tumoricidal activity. Governed by its concentration, spatial localization and exposure kinetics, NO acts as a master regulatory signal that orchestrates multiple components of the tumour ecosystem (Figure 1). This paradigm shift raises a compelling biological question: can NO be successfully repositioned from a fleeting cytotoxic weapon into a programmable architect of the TME?

**FIGURE 1:**
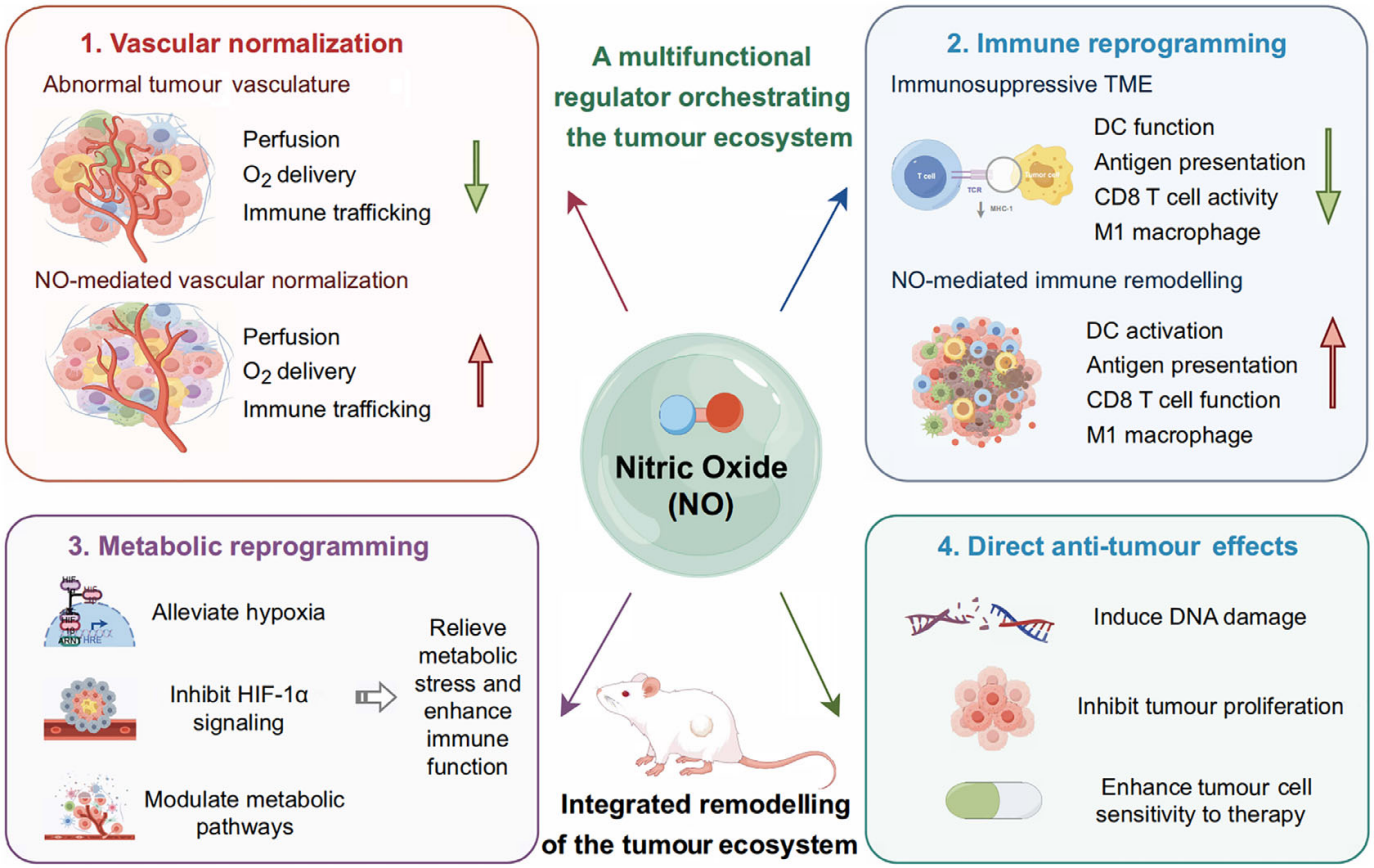
Nitric oxide as an ecosystem regulator in cancer immunotherapy.

Despite its immense therapeutic potential, the clinical translation of NO has long been thwarted by intrinsic pharmacological hurdles, notably its transient half‐life, rapid diffusion kinetics, and the impossibility of achieving sustained, localized intratumoral production. Traditional delivery modalities—such as small‐molecule donors and advanced nanomaterials—have sought to mitigate these limitations, yet they consistently fail to achieve precise spatiotemporal control.^2^ Recent breakthroughs in synthetic biology offer a transformative solution by converting NO from a transient chemical mediator into a programmable therapeutic cue.

The development of engineered microorganisms capable of sustained intratumoral NO production provides a compelling proof‐of‐concept for this strategy.^3^, ^4^ By rewiring bacterial metabolism towards endogenous NO synthesis, engineered microbes can function as localized ‘living factories’ that continuously generate NO within the tumour niche. Rather than relying on direct tumour cell lysis, these NO‐producing bacteria reshape the tumour ecosystem through the coordinated, multi‐target regulation of both vascular and immune components.^5^

A cornerstone of this NO‐mediated remodeling is vascular normalization.^6^ Tumour vasculature is notoriously characterized by structural anomalies, chaotic perfusion and impaired immune cell trafficking, constituting a formidable physical and biochemical barrier to immunotherapy. Localized NO production promotes vascular maturation and restores endothelial integrity, thereby lowering barriers to immune cell accessibility. By re‐engineering a functional vascular network and upregulating endothelial adhesion molecules, NO facilitates the robust recruitment and infiltration of cytotoxic lymphocytes into deep tumour beds. This mechanism challenges the conventional view of tumour vasculature merely as a passive delivery barrier, highlighting blood vessels instead as active regulators of antitumour immunity.

Beyond vascular regulation, NO profoundly reprograms the tumour immune landscape. A suppressive TME—harbouring dysfunctional antigen‐presenting cells, regulatory myeloid subsets, and exhausted T cells—collectively thwarts endogenous and therapeutic immune responses. NO‐mediated remodeling overcomes these obstacles by promoting dendritic cell activation, boosting antigen presentation, skewing macrophage polarization towards an immunostimulatory phenotype, and depleting suppressive myeloid populations. These concerted alterations convert immune‐excluded environments into immune‐responsive niches, thereby sensitizing refractory tumours to ICB.

Crucially, the capacity of NO to remodel the tumour ecosystem illuminates a broader therapeutic principle: successful immunotherapy relies not only on empowering immune effectors, but also on reconstructing the ecological conditions required for sustained immune function. In this context, NO emerges as a unique class of therapeutic regulator that simultaneously tunes vascular biology, awakens immune surveillance and potentiates treatment responsiveness.

Nevertheless, several critical challenges must be addressed before NO‐based ecosystem engineering can achieve clinical reality. First, precise control over NO dosage and kinetics is paramount; insufficient levels fail to drive TME remodeling, whereas excess NO may provoke off‐target toxicity or unintended immunosuppression. Future strategies synergizing synthetic biology, responsive genetic circuits and advanced biomaterials will be vital for achieving high‐precision NO regulation.^7^ Second, NO‐mediated remodeling warrants rigorous evaluation in combination with emerging immunotherapies—including cancer vaccines, adoptive cellular therapies, and immune agonists—where TME reprogramming can surmount existing spatial and cellular bottlenecks. Finally, clinical translation will necessitate stringent assessments of biosafety, controllability and scalable manufacturing.^8^

Collectively, these advances advocate for a fundamental re‐evaluation of NO: no longer viewed merely as a reactive cytotoxic species, it stands as a programmable architect of tumour ecosystems. As the future of cancer immunotherapy increasingly pivots toward engineering the tumour environment itself, NO‐mediated microenvironment remodeling offers a powerful, paradigm‐shifting strategy to bridge the gap between immune activation and durable clinical responses.

## Data Availability

The data that support the findings of this study are available from the corresponding author upon reasonable request.

## References

[1] Binnewies M , Roberts EW , Kersten K , et al. Understanding the tumor immune microenvironment (TIME) for effective therapy. Nat Med. 2018;24(5):541‐550. doi:10.1038/s41591‐018‐0014‐x 29686425 10.1038/s41591-018-0014-x PMC5998822

[2] Tang Y , Li Q , Zhou Z , et al. Nitric oxide‐based multi‐synergistic nanomedicine: an emerging therapeutic for anticancer. J Nanobiotechnology. 2024;22(1):674. doi:10.1186/s12951‐024‐02929‐z 39497134 10.1186/s12951-024-02929-z PMC11536969

[3] Chowdhury S , Castro S , Coker C , Hinchliffe TE , Arpaia N , Danino T . Programmable bacteria induce durable tumor regression and systemic antitumor immunity. Nat Med. 2019;25(7):1057‐1063. doi:10.1038/s41591‐019‐0498‐z 31270504 10.1038/s41591-019-0498-z PMC6688650

[4] Gurbatri CR , Arpaia N , Danino T . Engineering bacteria as interactive cancer therapies. Science. 2022;378(6622):858‐864. doi:10.1126/science.add9667 36423303 10.1126/science.add9667 PMC10584033

[5] Xu S , Zhang T , Song Y , et al. Sustained nitric oxide production by engineered *E. coli* remodels the tumor microenvironment and potentiates immunotherapy. Nat Biotechnol. 2026. doi:10.1038/s41587‐026‐03054‐y 10.1038/s41587-026-03054-y 41851305

[6] Choi Y , Jung K . Normalization of the tumor microenvironment by harnessing vascular and immune modulation to achieve enhanced cancer therapy. Experiment Mol Med. 2023;55(11):2308‐2319. doi:10.1038/s12276‐023‐01114‐w 10.1038/s12276-023-01114-w PMC10689787 37907742

[7] Zhou T , Wu J , Tang H , et al. Enhancing tumor‐specific recognition of programmable synthetic bacterial consortium for precision therapy of colorectal cancer. NPJ Biofilm Microbio. 2024;10(1):6. doi:10.1038/s41522‐024‐00479‐8 10.1038/s41522-024-00479-8 PMC10799920 38245564

[8] Zhu B , Yin H , Zhang D , et al. Synthetic biology approaches for improving the specificity and efficacy of cancer immunotherapy. Cell Mol Immunol. 2024;21(5):436‐447. doi:10.1038/s41423‐024‐01153‐x 38605087 10.1038/s41423-024-01153-x PMC11061174

